# Targeted suppression of AR-V7 using PIP5K1α inhibitor overcomes enzalutamide resistance in prostate cancer cells

**DOI:** 10.18632/oncotarget.11757

**Published:** 2016-08-31

**Authors:** Martuza Sarwar, Julius Semenas, Regina Miftakhova, Athanasios Simoulis, Brian Robinson, Anette Gjörloff Wingren, Nigel P. Mongan, David M. Heery, Heather Johnsson, Per-Anders Abrahamsson, Nishtman Dizeyi, Jun Luo, Jenny L. Persson

**Affiliations:** ^1^ Division of Experimental Cancer Research, Department of Translational Medicine, Lund University, Clinical Research Centre, Malmö, Sweden; ^2^ Department of Genetics, Kazan Federal University, Kazan, Russia; ^3^ Department of Clinical Pathology and Cytology, Skåne University Hospital, Malmö, Sweden; ^4^ Department of Pathology, Weill Cornell Medical College, New York, NY, USA; ^5^ Faculty of Health and Society, Department of Biomedical Science, Malmö University, Malmö, Sweden; ^6^ Faculty of Medicine and Health Sciences, School of Veterinary Medicine and Sciences, University of Nottingham, Nottingham, United Kingdom; ^7^ School of Pharmacy, University of Nottingham, Nottingham, United Kingdom; ^8^ Department of Bio-Diagnosis, Beijing Institute of Basic Medical Sciences, Beijing, China; ^9^ Division of Clinical Urology, Department of Translational Medicine, Lund University, Clinical Research Centre, Malmö, Sweden; ^10^ Department of Urology, the James Buchanan Brady Urological Institute, Johns Hopkins University School of Medicine, Baltimore, MD, USA; ^11^ Department of Molecular Biology, Umeå University, Sweden

**Keywords:** prostate cancer metastasis, enzalutamide resistance, lipid kinase inhibitor, AR-V7, PIP5K1α

## Abstract

One mechanism of resistance of prostate cancer (PCa) to enzalutamide (MDV3100) treatment is the increased expression of AR variants lacking the ligand binding-domain, the best characterized of which is AR-V7. We have previously reported that Phosphatidylinositol-4-phosphate 5-kinase alpha (PIP5Kα), is a lipid kinase that links to CDK1 and AR pathways. The discovery of PIP5Kα inhibitor highlight the potential of PIP5K1α as a drug target in PCa. In this study, we show that AR-V7 expression positively correlates with PIP5K1α in tumor specimens from PCa patients. Overexpression of AR-V7 increases PIP5K1α, promotes rapid growth of PCa in xenograft mice, whereas inhibition of PIP5K1α by its inhibitor ISA-2011B suppresses the growth and invasiveness of xenograft tumors overexpressing AR-V7. PIP5K1α is a key co-factor for both AR-V7 and AR, which are present as protein-protein complexes predominantly in the nucleus of PCa cells. In addition, PIP5K1α and CDK1 influence AR-V7 expression also through AKT-associated mechanism dependent on PTEN-status. ISA-2011B disrupts protein stabilization of AR-V7 which is dependent on PIP5K1α, leading to suppression of invasive growth of AR-V7-high tumors in xenograft mice. Our study suggests that combination of enzalutamide and PIP5K1α may have a significant impact on refining therapeutic strategies to circumvent resistance to antiandrogen therapies.

## INTRODUCTION

Although considerable progress is being made to improve therapy of PCa, one-third of treated PCa patients will experience disease recurrence and will progress into castration-resistant PCa (CRPC), which are no longer responsive to anti-androgen therapies [1–4]. One mechanism of resistance to antiandrogen treatment is the increased expression of AR variants lacking the ligand binding-domain, the best characterized of which is AR-V7 [5–7]. AR-V7 is a constitutive active variant lacking the LBD encoded by exon 4-8, owing to utilization of a cryptic exon [7]. AR-V7 expression is significantly higher in CRPC specimens than in specimens from hormone-naïve PCa and is associated with worse clinical outcome, suggesting an important role of AR-V7 in development of CRPC [8, 9].

Enzalutamide inhibits recruitment of AR coactivators and prevents AR nuclear localization [10]. Although enzalutamide is beneficial for patients with metastatic CRPC, giving an increasing in survival [11, 12], a rapid development of resistance in PCa patients to enzalutamide treatment remain to be a major clinical challenge [11]. The increased level of AR-V7 transcript was detected in circulating tumor cells from PCa patients who developed enzalutamide resistance [13]. It is believed that AR-V7 lacking the LBD evades enzalutamide, which acts via the LBD [11, 14]. However, the AR-V7-mediated cellular mechanisms in resistance to enzalutamide remain poorly understood.

Phosphatidylinositol 4-phosphate 5-kinase alpha (PIP5K1α) and its phospholipid product PIP2 have emerged as a new class of predictive biomarkers and drug targets in prostate cancer [15]. PIP5K1α is a lipid kinase similar to PI3K and acts on PI3K/AKT/PTEN pathways, and thereby regulates cell survival and migration [16]. PIP5K1α produces PIP2, a major substrate for triggering PI3K/AKT activity which is also regulated by PTEN to turn off the over-activated AKT [17]. Related to this, alterations in AR and PI3K/AKT/PTEN signaling complexes cooperatively contribute to PCa progression [18, 19]. We recently described a selective PIP5K1α inhibitor, ISA-2011B, which blocks the PI3K/AKT pathway by decreasing AKT phosphorylation at Serine 473 (pAKT S473), and suppresses growth of aggressive tumors in xenograft mice [15, 20, 21]. Further, we have shown that PIP5K1α cross-interacts with AR, and ISA-2011B significantly inhibits AR and PSA expression and induced apoptotic cell death in LNCaP cells[15].

CDK1 is the most essential kinase in CDK family, and is sufficient to drive cell division cycle in mammalian cells [22]. CDK1 is in complex with cyclin B1 and cyclin A, and is a component of the PI3K/AKT kinase network. This network is upregulated by extracellular chemokines in PCa cells during invasion [23]. Indeed, CDK1 phosphorylates AR and thereby enhances AR activity during progression of castration resistant PCa [24]. However, the precise role of CDK1 in driving PCa progression is unclear.

In the present study, we have studied the role of AR-V7 and its functional association with PIP5K1α and CDK1 in PCa progression using PCa cell lines and xenograft mouse models. We have further investigated whether the PIP5K1α inhibitor, ISA-2011B might suppress the growth of AR-V7-overexpressing tumors and selectively block the deregulated AR-V7 pathways in xenograft mouse models. Our study identifies novel cooperative mechanisms involving PIP5K1α, AR-V7, CDK1 and AR, which drive tumor progression and contribute to enzalutamide resistance. Our findings provide new information to guide the development of targeted therapeutic combinations for treatment of invasive castration-resistant PCa.

## RESULTS

### Expression of AR-V7 and PIP5K1α in primary PCa and in PCa metastatic specimens

**Figure 1:**
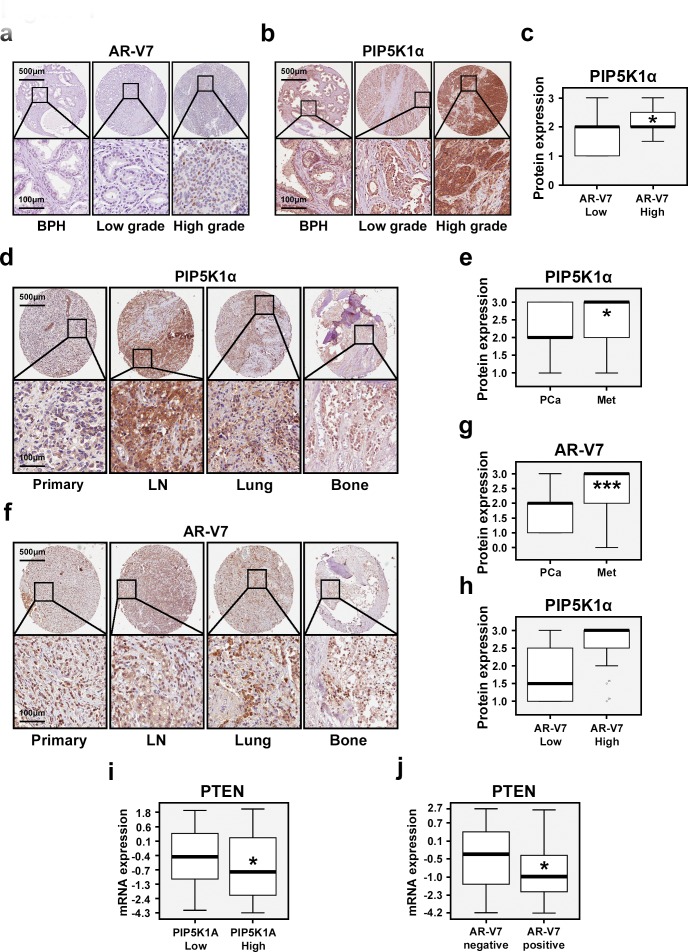
Evaluation of the clinical importance of AR-V7 and its correlation with PIP5K1α in PCa patients Immunohistochemical analysis of AR-V7 and PIP5K1α expression on the TMAs that contain BPH, PCa specimens and metastatic PCa tissues. (**a** and **b**). Representative microphotographs show the expression of AR-V7 and PIP5K1α in BPH, low Gleason score- and high Gleason score-tumors. Gleason score lower than 6 was defined as “Low grade”. Tumors with Gleason score greater than 8 were defined as “High grade”. Scale bars are indicated. (**c**). Box-plot quantitative comparison shows that PIP5K1α expression is increased in AR-V7-high group (n=9) compared with that in AR-V7-low group (n=36). *p*= 0.045. (**d** and **f**). Representative microphotographs show the expression of PIP5K1α and AR-V7 in primary tumor tissues and metastatic tissues including lymph node (LN), lung, and bone. (**e** and **g**). Box-plot quantitative comparisons of PIP5K1α and AR-V7 expression between primary cancer and metastatic cancer specimens are shown. For PIP5K1α, primary PCa: PCa (n=61), and the metastatic tissues: Met (n=41) were compared, *p*= 0.014. For AR-V7, primary PCa (n=63) and metastases (n=40) were compared, *p*< 0.001. (**h**). Box-plot quantitative comparison shows that PIP5K1α expression is increased in AR-V7-high group (n=6) compared with AR-V7-low group (n=50) in metastatic tissues. *p*= 0.056. (**i**). Box-plot quantitative comparison shows that PTEN mRNA expression is down-regulated in PIP5K1A-High group (n=127) compared with PIP5K1A-Low group (n =163), *p*= 0.011. (**j**) Box-plot quantitative comparison shows that PTEN mRNA expression is down-regulated in PCa tissues that have AR-V7 expression (AR-V7 positive, n =70), compared with PCa tissues without AR-V7 expression (AR-V7 negative, n = 220), *p*= 0.023.

To evaluate clinical importance of AR-V7 and its correlation with PIP5K1α expression in PCa patients, the first sets of tissue microarrays (TMAs) consisting of begin prostate hyperplasia (BPH) (n=48), primary PCa (n=65), and PCa metastatic tissues (n=43) were immuno-stained with antibodies against AR-V7 and PIP5K1α. A second TMA that contained biopsies of BPH and cancer tissues from 48 PCa patients was also examined for AR-V7 and PIP5K1α expression. AR-V7 expression was predominantly nuclear, whereas PIP5K1α expression was present in both cytoplasm and nucleus of cancer cells (Figure 1a and 1b). PIP5K1α expression was increased in tumors with high level of AR-V7 compared with that of tumors with lower AR-V7 expression (*p*=0.045, Figure 1c). Metastatic lesions in lymph nodes, lungs and bones displayed increased PIP5K1α compared with that of primary PCa (*p*=0.014; Figure 1d and 1e). Similarly, AR-V7 expression was also significantly higher in metastasis tissues compared with that in primary PCa (*p*<0.001, Figure 1f and 1g). Thus, elevated level of AR-V7 positively correlates with the increased PIP5K1α expression in primary PCa tissues. A trend of the correlation between AR-V7 and PIP5K1α was also observed in metastatic tissues (*p*=0.056, Figure 1h). We next examined PIP5K1A expression in AR-V7 positive or negative primary PCa tissues (n=333) using the National Cancer Institute database [25]. Although there was no statistically significant correlation between AR-V7 and PIP5K1A mRNA expression in primary PCa of this patient cohort, we observed that PTEN expression was decreased in tumors with higher PIP5K1A as compared to those with lower PIP5K1A (*p*=0.011, Figure 1i). Similarly, PTEN expression was significantly decreased in AR-V7-positive tumors as compared to that in AR-V7-negative tumors (*p*= 0.023, Figure 1j). These data suggest that expression of AR-V7 and PIP5K1A correlates with PTEN-status in PCa patients.

### Elevated expression of AR-V7 contributes to increased PIP5K1α and tumor progression

**Figure 2:**
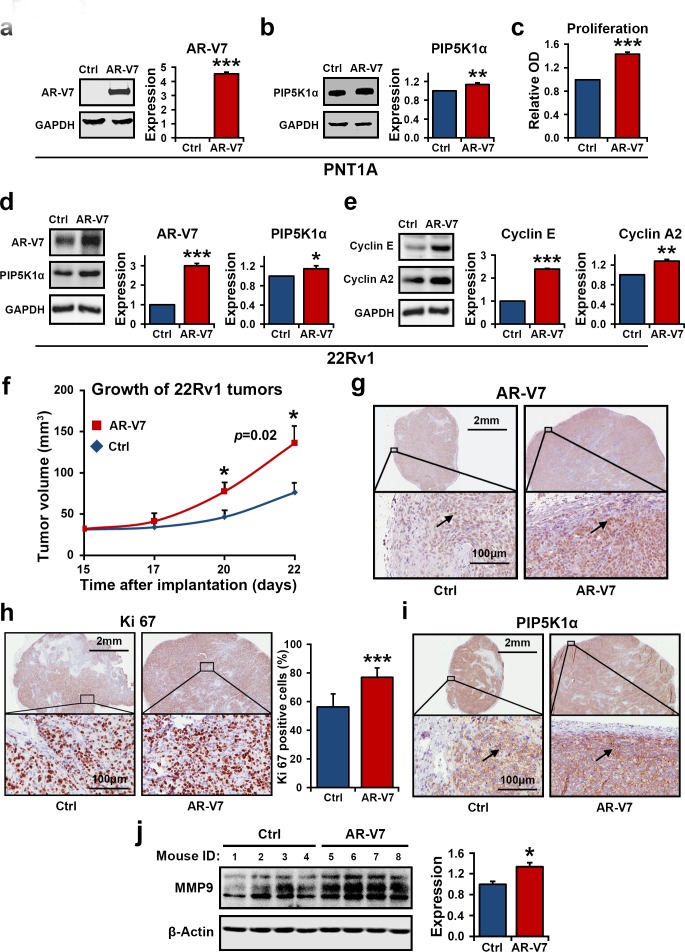
The effect of elevated expression of AR-V7 on growth of tumor cells *in vitro* and *in vivo* (**a** and **b**). Immunoblots show the effect of AR-V7 overexpression on PNT1A cells. Quantification of the blots from three independent experiments is shown in the right panel. The mean expression of AR-V7 in cells transfected with pEGFP control vector (Ctrl) was 0 and the mean value in cells transfected with pEGFP-AR-V7 (AR-V7) was 4.53 (difference =4.53; 95% CI= 4.28 to 4.78, *p*<0.001). The mean expression of PIP5K1α in cells transfected with pEGFP was set as 1, and the mean value in cells transfected with pEGFP-AR-V7 was 1.13 (difference= 0.13, 95% CI=1.06 to 1.21, *p*=0.006). (**c**). The effect of AR-V7 expression on proliferation of PNT1A cells was assessed using the non-radioactive MTS proliferation assay. Mean absorbance for control PNT1A cells and AR-V7 expressing cells were 0.23 and 0.34 respectively (difference=0.11; 95% CI=32 to 35, *p*=0.002). (**d** and **e**). Immunoblots show the effect of AR-V7 overexpression on 22Rv1 cells. Overexpression of AR-V7 increases expression of PIP5K1α, cyclin E and cyclin A2 in 22Rv1cells. For AR-V7, *p*<0.001 and for PIP5K1α, *p*=0.041. The mean expression of cyclin E1 in control and AR-V7 overexpressing cells were 1 and 2.39 respectively (difference =1.39, 95% CI= 2.35 to 2.44, *p*<0.001). The mean cyclin A2 expression in control and AR-V7 overexpressing cells were 1 and 1.28 respectively (difference=0.28, 95% CI=1.21 to 1.35, *p*=0.002). The above data are presented as average of three or four independent experiments (±SD). *p<0.05, **p<0.01 and ***p<0.001 are indicated. (**f**). Growth curves of 22Rv1 tumor xenografts expressing pEGFP-AR-V7 or pEGFP control vector. Difference in mean tumor volumes was calculated. *p*=0.02 on day 22 is indicated. (**g**, **h** and **i**) Tumors from each group were collected at the end of experiment, and were subjected to immunohistochemical analysis. Representative microphotographs show the expression of AR-V7, Ki-67 and PIP5K1α in xenograft tumors. Mean percentages of Ki-67-positive cells in control and AR-V7 overexpressing tumors were 56.17%, and 77.05% respectively (difference=20.89%, 95% CI=72.84 to 81.26; P< 0.001). (**j**) Immunoblots show that mean expression of MMP9 in control and AR-V7 overexpressing tumors was 36597.43 vs. 48960.61 (difference=12363.17, 95% CI=43375.74 to 54545.47, *p*=0.012) (n= 4 mice/per group).

We further characterized the biological consequences of AR-V7 overexpression in PCa cells and the underlying molecular mechanisms. We introduced overexpression of AR-V7 by transfecting non-malignant PNT1A cells, which do not express endogenous AR-V7, with pEGFP-AR-V7 or pEGFP control vectors. Expression of AR-V7 led to an increase in PIP5K1α expression in PNT1A cells, as measured by densitometric quantification of the blots from three independent experiments using Image J (for AR-V7 overexpression, *p*<0.001; for PIP5K1α, *p*=0.006, Figure 2a and 2b). This coincided with the increased proliferative activity of PNT1A cells that expressed AR-V7 vector as compared to the cells that expressed control vector (*p*=0.002, Figure 2c).

Since 22Rv1 cells represent a cellular model of AR-V7 expressing CRPC [14], we therefore examined the effect of elevated expression of AR-V7 on PIP5K1α in malignant 22Rv1 cells, which harbor AR-V7 and display robust growth under castration condition. Consistent with the results observed in PNT1A cells, overexpression of AR-V7 in 22Rv1 cells significantly increased PIP5K1α expression (For AR-V7 overexpression, p<0.001; for PIP5K1α, *p*=0.041, Figure 2d), also led to a significant increase in the levels of cyclin E (p<0.001) and cyclin A2 (p<0.002), both key determinants of cell proliferation (Figure 2e).

We further assessed whether 22Rv1 cells overexpressing AR-V7 may have gained invasive feature by using *in vivo* xenograft mouse model. To this end, 22Rv1 cells expressing AR-V7 or control vector were implanted subcutaneously into the nude mice. Multiple tumors were observed in all xenograft mice received 22Rv1 cells overexpressing AR-V7. The mean tumor volume overexpressing AR-V7 was significantly increase by 78% as compared with the controls (mean volume for pEGFP-control tumors =76.15 mm^3^, mean volume for pEGFP-AR-V7 tumors, the largest one among multiple tumors=136.02 mm^3^, difference=59.87 mm^3^; 95% CI=96.32-175.73; n=6 mice per group, *p*=0.02, Figure 2f). Thus, 22Rv1 cells overexpressing AR-V7 gained increased ability to grow into multiple tumors as compared with the controls. Immuno-histochemical analysis of the xenograft tumors revealed a remarkable increase in nuclear AR-V7 expression in tumors derived from 22Rv1 cells expressing AR-V7 (Figure 2g). Tumors derived from AR-V7-expressing cells also displayed higher proliferative activity, as measured by Ki67-staining (*p* < 0.001), and showed enhanced nuclear PIP5K1α expression (Figure 2h and 2i). The matrix metalloproteinase (MMP9), a key factor that promotes tumor metastasis, was significantly higher in tumors derived from AR-V7-expressing cells than in the controls as determined by immunoblot analysis (*p*=0.012, Figure 2j). Taken together, AR-V7 promotes growth of PCa and induces expression of PIP5K1α in the nucleus. This suggests a mechanistic link between AR-V7, PIP5K1α and PCa progression.

### PIP5K1α Inhibitor ISA-2011B Suppresses PCa Tumor Growth in Xenograft Mouse Models

**Figure 3:**
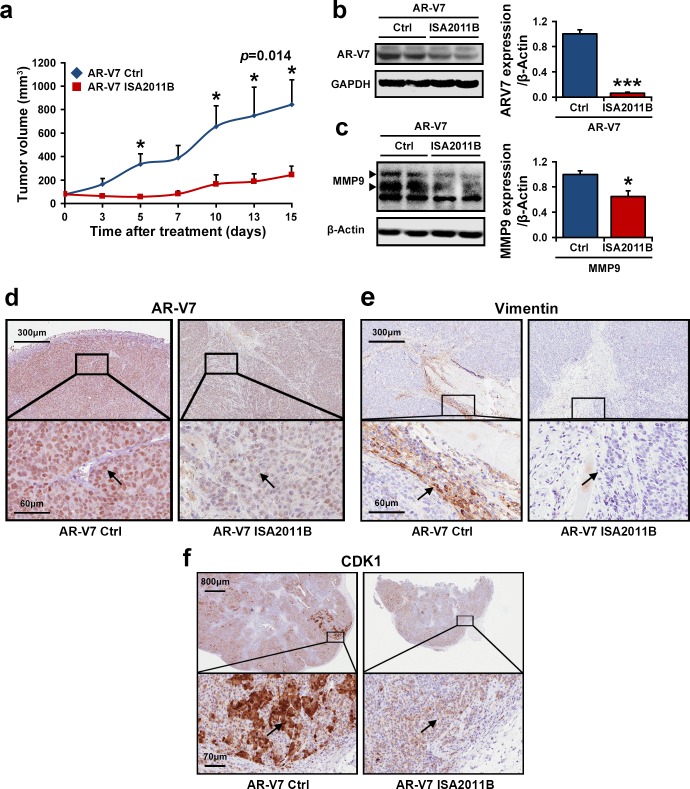
The inhibitory effect of PIP5K1α inhibitor ISA-2011B on growth of 22Rv1 xenograft tumors with elevated expression of PIP5K1α and AR-V7 *in vivo* (**a**). Growth of tumor xenografts overexpressing AR-V7 that were treated with vehicle (AR-V7 Ctrl) or ISA-2011B at 40 mg/kg (AR-V7 ISA2011B). Treatment ended on day 15. Mean tumor volumes and upper 95% confidence intervals are shown. Mean tumor volume of vehicle control group= 844.12 mm^3^, ISA-2011B treated group = 243.77 mm^3^ (difference = 600.35 mm^3^; 95% CI=96.09-391.44; *P=0.014 on day 15. (**b** and **c**). Tumors from each group were collected at the end of experiment. Immunoblots show the expression of AR-V7 and MMP9 in tumors from two groups. Mean expression of AR-V7 in vehicle treated vs. ISA-2011B treated was 6378.85 and 406.72 (difference=5972.13, 95% CI=214.34 to 599.91, *p*=0.002; mean MMP9 expression in vehicle treated vs. ISA-2011B treated was 48960.61, and 31866.49 (difference=17094.12, *p*=0.018). (**d**, **e** and **f**). Representative microphotographs of immunohistochemical analysis show the expression and cellular localization of AR-V7, CDK1 and Vimentin in vehicle treated tumors *vs.* ISA-2011B treated tumors.

Given the mechanistic link we have established between AR-V7 and PIP5K1α, we therefore hypothesized that AR-V7 cooperates with PIP5K1α to contribute to tumor growth, thus treatment of tumors overexpressing AR-V7 with ISA-2011B, a selective PIP5K1α inhibitor may suppress the tumor growth through blocking the deregulated AR-V7 pathways. To investigate this, we established 22Rv1 tumors overexpressing AR-V7 in mice, and then treated these mice bearing AR-V7-overexpressing tumors (100-150 mm^3^ in mean volumes) with ISA-2011B or vehicle control. After two weeks of treatment, the mean tumor volumes in mice treated with ISA-2011B was 3.5-fold smaller compared with those treated with vehicle control (mean volume of tumors for vehicle-treated controls = 844.12 mm^3^, and for ISA-2011B-treated = 243.77 mm^3^, difference = 600.35 mm^3^; 95% CI=96.09-391.44; *p*=0.014, Figure 3a). We next examined the effect of ISA-2011B-treatment on AR-V7 and the key factors that promote tumor invasiveness in the xenograft tumors. Remarkably, AR-V7 expression in tumors from the ISA-2011B-treated group was decreased by 93% compared to the vehicle-treated controls (*p*=0.002, Figure 3b), and a significant decrease in MMP9 expression was also observed as determined by immunoblot analysis (*p*=0.018, Figure 3c). Immunohistochemical analysis further revealed a reduction in AR-V7 expression in both nucleus and cytoplasm of the tumor cells (Figure 3d). Similarly, both nuclear and cytoplasmic expression of CDK1 was also decreased in ISA-2011B-treted tumors as compared with the controls (Figure 3e). ISA-2011B-treatment also led to an inhibition in the expression of vimentin, a bio-marker for metastatic tumors, in the infiltrating tumor cells at the invasive front (Figure 3f). Thus, inhibition of PIP5K1α suppresses the growth and invasiveness of AR-V7-overexpressing tumors.

### Association of AR-V7, PIP5K1α and CDK1 in subcellular compartments

**Figure 4:**
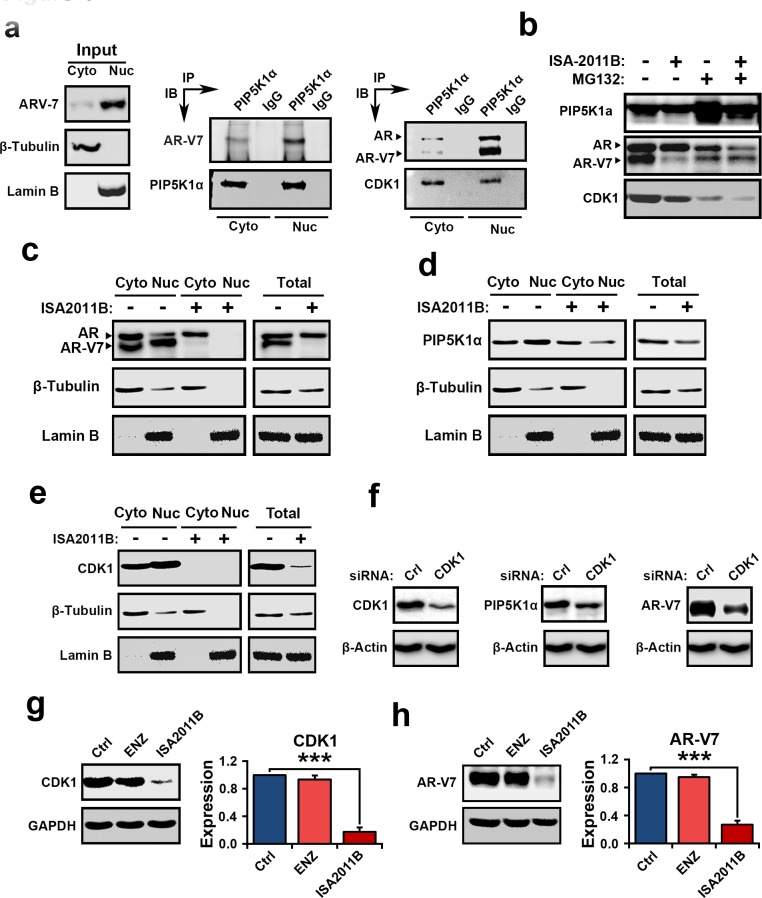
Evaluation the interconnection among AR-V7, PIP5K1α and CDK1 in the nuclear and cytoplasmic compartments of 22Rv1 cells and the mechanisms of the action of ISA2011B on AR-V7 (**a**). Cytoplasmic (Cyto) and nuclear (Nuc) fractions were separated from 22Rv1 cells and were subjected to immunoprecipitation (IP) assay. Antibody to PIP5K1α was used to pull down the immunocomplexes, and antibody to IgG was used as a negative control. Antibodies against PIP5K1α, AR-V7, AR and CDK1 were used for immunoblot analysis (IB). The cell lysates from cytoplasmic and nuclear fractions were used as “Input” controls as indicated in the left panel. β-tubulin and lamin B were used as controls for the cytoplasmic *vs*. nuclear fractions. (**b**). Evaluation of ISA-2011B-induced degradation of AR-V7 protein at PIP5K1α-dependent fashion using immunoblot analysis. Expression of PIP5K1α, AR-V7, AR and CDK1 was examined in control 22Rv1 cells, and in cells treated with ISA-2011B or MG132 alone, or ISA-2011B and MG132 together. (**c**, **d** and **e**). The effect of ISA-2011B on the subcellular expression of AR-V7, AR, PIP5K1α and CDK1 in 22Rv1 cells. The representative blots from the same membrane is shown. The expression of these proteins in cytoplasmic (cyto) and nuclear (nuc) compartments of 22Rv1 cells treated with vehicle control (−) or ISA-2011B (ISA-2011B+). Total cell lysates were used as controls (Total). The blots of β-tubulin and Lamin B used as controls were from the same detection as shown in b, c and d. (**f**). CDK1 was depleted by transfecting 22Rv1 cells with CDK1 siRNA or scramble control (Ctrl). The effect of CDK1 knockdown on PIP5K1α and AR-V7 in 22Rv1 cells is analyzed using immunoblot analysis. The representative immunoblots from the same membrane were used. The blots of β-tubulin used as controls were from the same detection as shown in f. (**g** and **h**). The side-by-side comparison of the effect of enzalutamide and ISA-2011B on CDK1 and AR-V7. Data is presented as mean of three independent experiments (±SD). ***p < 0.001.

The above results suggest that AR-V7, PIP5K1α and CDK1 may cooperatively promote tumor progression. Immunoprecipitation assays of the nuclear and cytoplasmic fractions of 22Rv1 cells further revealed that AR-V7 formed complexes with PIP5K1α in the nucleus and cytoplasmic compartments (Figure 4a). PIP5K1α in turn also formed complexes with CDK1 and AR in both nuclear and cytoplasmic compartments of 22Rv1 cells (Figure 4a). Thus, AR-V7 may physically interact with PIP5K1α, and CDK1 through formation of protein-protein complexes. Since AR-V7 forms proteins complexes with PIP5K1α, we next investigated whether a decrease in AR-V7 expression induced by ISA-2011B might be a result of disruption of AR-V7/PIP5K1α complexes. To this end, we treated 22Rv1 cells with ISA-2011B, MG132, a proteasome inhibitor alone or a combination of ISA-2011B and MG132 together. Expression of PIP5K1α was remarkably increased in 22Rv1 cells treated with MG132 (Figure 4b), suggesting that MG132 prevented proteasome-dependent degradation of PIP5K1α. ISA-2011B was unable to inhibit PIP5K1α expression in the presence MG132 (Figure 4b). This suggests that ISA-2011B inhibits PIP5K1α expression by disrupting its protein stabilization. Interestingly, MG132 treatment had no effect on AR-V7 protein stability, however, in the presence of MG132, ISA-2011B was unable to supress AR-V7 expression (Figure 4b). MG132 did not appear to have pronounced effect on protein stabilization of full-length AR or CDK1 expression (Figure 4b). Thus, ISA-2011B disrupts stabilization of PIP5K1α and AR-V7/PIP5K1α complexes, leading to a decrease in AR-V7 protein expression. This data suggests that ISA-2011B-induced effect on AR-V7 is specifically dependent on PIP5K1α-associated pathways. Next, we examined the effect of ISA-2011B on the expression and subcellular distribution of AR-V7, PIP5K1α, CDK1 and AR in 22Rv1 cells. Inhibition of PIP5K1α by ISA-2011B led to a remarkable reduction in AR-V7 and CDK1 in both nucleus and cytoplasm of 22Rv1 cells (Figure 4c, 4d and 4e). ISA-2011B treatment also abolished AR expression in the nucleus, without depleting the cytoplasmic AR (Figure 4c). It has been shown that CDK1 is able to phosphorylate AR and is believed to activate AR activity during progression of castration resistant PCa[24]. To define the functional link between CDK1 and AR-V7, we examined effect of inhibition of CDK1 on AR-V7. Inhibition of CDK1 via siRNA-mediated knockdown led to a remarkable reduction in AR-V7 expression and a concomitant decrease in PIP5K1α expression in 22Rv1 cells (Figure 4f). Similarly, inhibition of PIP5K1α by ISA-2011B significantly decreased CDK1 (*p<0.001,* Figure 4g) and AR-V7 in 22Rv1 cells (*p<0.001,* Figure 4h), while blocking AR signaling using enzalutamide had no inhibitory effect on CDK1 and *AR-V7* (Figure 4g and 4h). Taken together, the above findings suggest that AR-V7 interacts with PIP5K1α and CDK1. Further, abnormal AR-V7 expression may be effectively inhibited by selectively blocking PIP5K1α using its inhibitor ISA-2011B.

### Treatment of 22Rv1cells with ISA-2011B reduces expression of AR-V7 signaling complex

**Figure 5:**
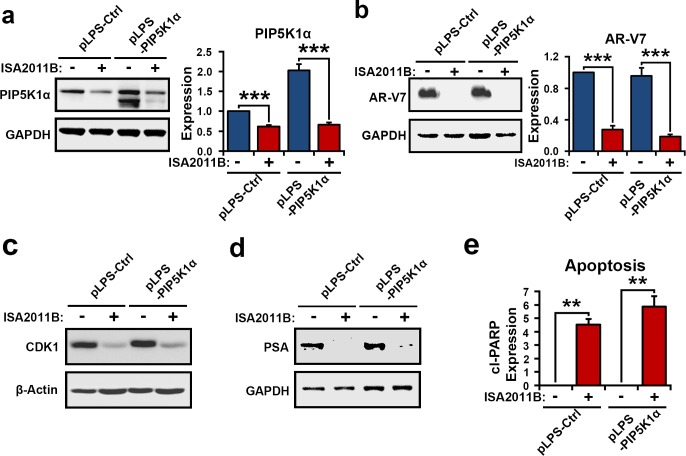
PIP5K1α overexpression and ISA-2011B on AR-V7 in 22Rv1 cells (**a** and **b**). Immunoblots show the effect of PIP5K1α overexpression on AR-V7 and the effect of ISA2011B on AR-V7 in 22Rv1 cells expressing PIP5K1α (pLPS-PIP5K1α) or control vector (pLPS-Ctrl). Mean expression of PIP51α in vehicle treated and ISA-2011B treated 22Rv1 cells was 1 vs. 0.55 (difference= 0.45 95% CI=0.47 to 0.64, p=0.001), mean AR-V7 in vehicle treated vs. ISA-2011B treated was 1 vs. 0.31 (difference=0.69, 95% CI=0.16 to 0.45, *p*<0.001). Mean PIP5K1α expression in PIP5K1α overexpressing cells treated with vehicle or ISA-2011B was 2.14 vs. 0.65 (difference=1.50, 95% CI=0.57 to 0.73; *p*<0.001). Mean AR-V7 expression in PIP51α overexpressing cells treated with vehicle or ISA-2011B was 0.98 vs. 0.23 (difference= 0.75, 95% CI= 0.15 to 0.31, *p*<0.001). (**c** and **d**). Immunoblots show the effect of ISA-2011B on expression of CDK1 and PSA in 22Rv1 cells expressing PIP5K1α or control vectors. (**e**). ISA2011B induced apoptosis in control (*p*=0.0043) and 22Rv1 cells overexpressing PIP5K1α (*p*=0.0085), as measured by the expression of the active PARP (cleaved CL. PARP).

PIP5K1α is a central factor that is highly expressed in metastatic PCa[15]. As mentioned in Figure 1, both PIP5K1α and AR-V7 were highly expressed in metastatic tissues from PCa patients. We therefore examined whether ISA-2011B might effectively induce apoptosis by specifically blocking PIP5K1α/AR-V7/CDK1 in PIP5K1α-overexpressing PCa cells. We introduced overexpression of PIP5K1α by transfecting of 22Rv1 cells with pLPS-EGFP-PIP5K1α or pLPS-EGFP control vector, followed by the treatment of the transfected cells with ISA-2011B or vehicle control. As expected, ISA-2011B was effective on both 22Rv1 cells overexpressing PIP5K1α (*p*<0.001) and control cells (*p*=0.001), and showed more pronounced inhibitory effect on cells overexpressing PIP5K1α (Figure 5a). ISA-2011B treatment led to a significant decrease in AR-V7 expression in both 22Rv1 cells overexpressing PIP5K1α (*p*<0.001) and control cells (*p*=0.001, Figure 5b). Overexpression of PIP5K1α greatly increased expression of CDK1 and PSA, while ISA-2011B treatment diminished CDK1 and PSA expression in PIP5K1α-overexpressing and control cells (Figure 5c and 5d). The inhibitory effect of ISA-2011B on AR-V7, CDK1 and PSA was co-incident with a significant induction in apoptosis, as determined by PARP activation, in 22Rv1 cells overexpressing PIP5K1α (*p*=0.0085) and in controls (*p*=0.0043, Figure 5e).

### The additive effect of combined treatment of 22Rv1 cells with ISA-2011B and enzalutamide

**Figure 6:**
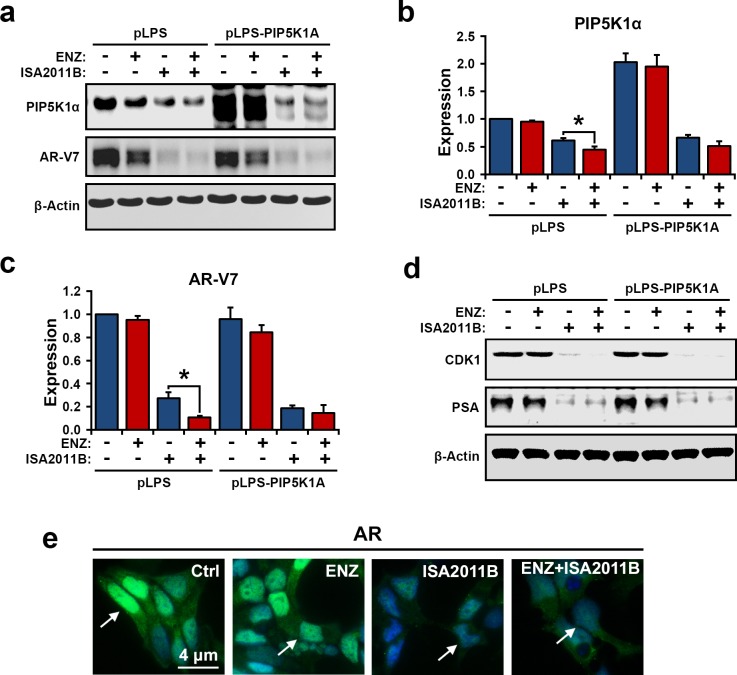
The effect of combination of enzalutamide and ISA-2011B on PIP5K1α, AR-V7 and AR in 22Rv1 cells overexpressing PIP5K1α or control vector (**a**). Immunoblot analysis shows the effect of enzalutamide, ISA-2011B and a combination of enzalutamide and ISA-2011B on 22Rv1 cells overexpressing PIP5K1α or control vector. (**b**). The combination of enzalutamide and ISA-2011B displays better inhibitory effect on PIP5K1α, as compared to ISA-2011B, *p=0.033. (**c**). The combination of enzalutamide and ISA-2011B displays better inhibitory effect on AR-V7, as compared to ISA-2011B, *p=0.036. Data is presented as average of three independent experiments (±SD). (**d**). The effect of enzalutamide and ISA-2011B alone or in combination on CDK1 and PSA in 22Rv1 cells overexpressing PIP5K1α or control vectors. (**e**). Representative immunofluorescent images show the expression and subcellular localization of AR in 22Rv1 cells overexpressing PIP5K1α that were treated with vehicle control, enzalutamide, ISA-2011B and combination of enzalutamide and ISA-2011B.

Enzalutamide targets ligand-binding domain of AR, therefore inhibits AR activity. However, enzalutamide has no inhibitory effect on AR-V7 in castration-resistant 22Rv1 cells [14]. In contrast to enzalutamide, ISA-2011B treatment significantly reduces AR-V7 expression in *in vitro* and *in vivo* PCa models as mentioned above. To test the therapeutic potentials of combination therapies of ISA-2011B and enzalutamide, we treated 22Rv1 cells overexpressing PIP5K1α or control vector with enzalutmide, ISA-2011B, or combination of enzalutamide and ISA-2011B. As expected, ISA-2011B treatment alone readily reduced expression of PIP5K1α, AR-V7, CDK1 and PSA (Figure 6a, 6b, 6c and 6d). However, the inhibitory effect of a combination of ISA-2011B and enzalutamide on PIP5K1α and AR-V7 expression was significantly greater than that for ISA-2011B alone (for PIP5K1α, *p=0.007*; for AR-V7, *p=0.07*, Figure 6b and 6c). Immunofluorescence analysis was performed to visualize the treatment effect on AR expression. Enzalutamide greatly reduced the cytoplasmic AR, whereas ISA-2011B decreased the nuclear AR in 22Rv1 cells. As expected, a combination of enzalutamide and ISA-2011B abolished both nuclear and cytoplasmic AR expression in 22Rv1 cells, showing a greater effect than the single agent (Figure 6e). These data suggest that combination treatment using enzalutamide and ISA-2011B has an additive effect and completely inhibits deregulated AR and AR-V7 mediated pathways.

### Treatment of metastatic cancer cell lines containing PTEN mutation with ISA-2011B and enzalutamide in combination

**Figure 7:**
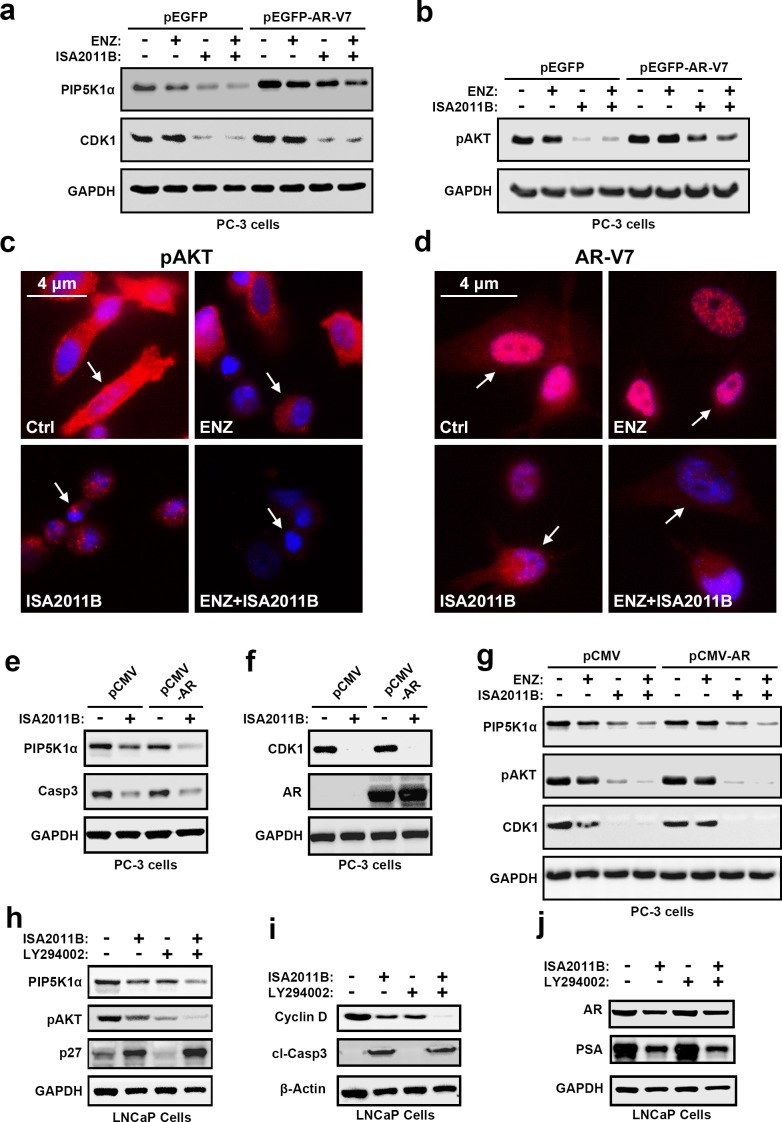
The interconnection among AR-V7, PIP5K1α, pAKT S473, CDK1 and AR signaling complexes in PCa cells harboring PTEN-mutations and the effect of combination of enzalutamide and ISA-2011B on PCa cells with PTEN mutations (**a** and **b**). The effect of AR-V7 overexpression on PC3 cells harboring PTEN-muations, and the effect of ISA-2011B, enzalutamide and combination of enzalutamide and ISA-2011B on PC3 cells expressing AR-V7. Immunoblots show the expression of PIP5K1α, CDK1 and pAKT S473. (**c** and **d**). Representative immunofluorescent images of subcellular localization of pAKT S473 and AR-V7 expression in PC3 cells expressing AR-V7 that were treated with vehicle control (Ctrl), enzalutamide, ISA-2011B and combination of enzalutamide and ISA-2011B (ENZ+ISA2011B). The cancer cells are indicated by the arrows. (**e** and **f**). The effect of ISA-2011B on PC3 cells expressing AR. Immunoblots show the expression of PIP5K1α, CDK1 and AR as well as caspase 3 as a marker for apoptosis in vehicle treated or ISA-2011B treated PC3 cells expressing control (PCMV) or AR expressing vectors (PCMV-AR). (**g**). The effect of enzalutamide, ISA-2011B and combination of enzalutamide and ISA-2011B on PC3 cells expressing AR. Immunoblots show the expression of PIP5K1α, pAKT S473 and CDK1. (**h**, **i** and **j**). The effect of ISA-2011B, LY294002 and combination of ISA-2011B and LY294002 on PIP5K1α, pAKT S473 in LNCaP cells. Immunoblots show the expression of PIP5K1α, pAKT S473, P27, AR, PSA and cyclin D1, as well as the activation of caspase-3 (cleaved caspase 3).

PIP5K1α acts upstream of PI3K/AKT pathways, and PTEN status is essential for controlling the proper activity of AKT[17]. We therefore next examined the functional relationship between ARV-7 and PIP5K1α in PCa cells lacking functional PTEN. To this end, PC-3 cells that do not express PTEN or AR, but express elevated level of pAKTS473, were transfected with pEGFP-AR-V7 or control vector. Aberrant AR-V7 expression led to an increase in PIP5K1α and a concomitant increase in pAKTS473 expression, as well as CDK1 on immunoblot analysis (Figure 7a and 7b). Immunoblot analysis was performed to assess the effect of enzalutamide, ISA-2011B, or a combination of ISA-2011B and enzalutamide on the expression of PIP5K1α, pAKTS473 and CDK1 in AR-V7-expressing PC3 cells. ISA-2011B and the combination of ISA-2011B and enzalutamide greatly inhibited PIP5K1α, pAKTS473 and CDK1 in AR-V7-expressing PC3 cells (Figure 7a and 7b). As expected, enzalutamide had no inhibitory effect on these proteins (Figure 7a and 7b). Immunofluorescence analysis indicated that ISA-2011B alone effectively reduced pAKTS473 levels in AR-V7-expressing PC3 cells (Figure 7c). ISA-2011B treatment effectively diminished the expression of AR-V7 in both nucleus and cytoplasm of PC3 cells (Figure 7c and 7d). We did not observe a statistically significant difference in pAKTS473 or AR-V7 inhibitions between the combination of ISA-2011B and enzalutamide as compared to ISA-2011B alone in AR-V7-expressing PC3 cells (Figure 7a and 7b). Thus, ISA-2011B alone is sufficient to inhibit AR-V7 and pAKT in PTEN- and AR-negative PCa cells expressing elevated AR-V7 and pAKTS473.

We next wanted to further study the relationship between AR and PIP5K1α/CDK1, and thereby gain a deeper understanding of the molecular mechanisms underlying the action of ISA-2011B on AR and AR-V7. We introduced AR expression by transfecting PC3 cells with pCMV-AR or pCMV control vectors, and subsequently treated AR-expressing PC3 cells with ISA-2011B, enzalutamide, or combination of ISA-2011B and enzalutamide. ISA-2011B and a combination of ISA-2011B and enzalutamide had pronounced inhibitory effect on PIP5K1α, pAKTS473, CDK1 in AR-expressing PC3 cells as compared to control PC3 cells (Figure 7e, 7f and 7g).

### The interconnection between PIP5K1α and pAKT S473

As mentioned above, ISA-2011B acts on multiple cellular pathways. We further validated its specificity on PIP5K1α-associated PI3K/AKT and AR signaling complexes by using PTEN-negative PCa cells expressing functional AR. We compared the effect of ISA-2011B with LY294002, an inhibitor of PI3K/AKT in PTEN-negative LNCaP cells containing functional AR, but expressing elevated pAKTS473. We treated LNCaP cells with ISA-2011B, LY294002 or a combination of ISA-2011B and LY294002. ISA-2011B or LY294002 treatment led greatly inhibited pAKTS473 and cyclin D1 expression (Figure 7h, 7i and 7j). A combination of ISA-2011B and LY294002 showed a greater effect, as compared to the single agent, to inhibit pAKTS473 and cyclin D1 expression in LNCaP cells (Figure 7h and 7i). This suggests that the action of ISA-2011B on PI3K/AKT pathways is similar to that of LY294002. However, in striking contrast to LY294002, ISA-2011B was able to inhibit AR and PSA expression, while LY294002 did not show any effect on AR or PSA expression (Figure 7j). A combination of ISA-2011B and LY294002 showed similar inhibitory effects as ISA-2011B alone on AR and PSA (Figure 7j), suggesting that LY294002 did not interfere with or augment ISA-2011B. In addition, LY294002 treatment reduced p27 expression, whereas ISA-2011B showed the opposite effect and increased expression of p27, a key cell cycle inhibitor to inhibit abnormal proliferation (Figure 7h). The effect of ISA-2011B on PIP5K1α-associated PI3K/AKT and AR signaling complexes was co-incident its action to induce apoptosis as measured by the induction of caspase-3 activity (Figure 7h). These data suggest that ISA-2011B acts as PI3K/AKT inhibitor similar to LY294002, but also has distinct role act as inhibitor of AR signaling complexes.

## DISCUSSION

Increasing evidence suggests that AR-V7 is involved in development of CRPC, bone metastasis and resistance to enzalutamide[5, 7, 8]. However, the precise mechanisms and co-factors of AR-V7 that act cooperatively to promote progression of CRPC are largely unknown. Further, no therapeutic compounds are currently available for treatment of patients with PCa resistance to enzalutamide. In the present study, we show that AR-V7 is highly expressed in metastatic tissues from PCa patients and its expression correlates with elevated level of PIP5K1α expression in tumor tissues from PCa patients. We further demonstrate that overexpression of AR-V7 promotes growth and invasiveness of castration-resistant 22Rv1 tumors in xenograft mice. Overexpression of AR-V7 significantly induces cyclin A2, cyclin E and MMP9 expression, suggesting that PIP5K1α/AR-V7 may act on these target genes, which are in accordance with their action on promoting growth and invasiveness of PCa *in vitro* and *in vivo*. Our findings provide evidence suggesting that AR-V7 is functionally associated with PIP5K1α.

**Figure 8:**
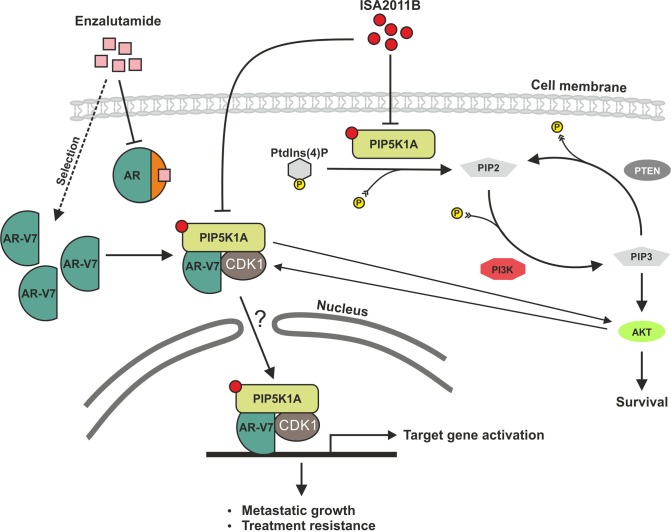
A schematic model shows that PIP5K1α acts on two parallel signaling pathways The model depicts PIP5K1α acts on two parallel pathways. PIP5K1α is the key kinase responsible for generation of PIP2. In turns, PIP2 serves as a precursor molecule required for PI3K activity and generation of PIP3, thus PIP5K1α acts upstream of PI3K/AKT as illustrated in our previous reported studies (Semenas et al., 2014) and the data presented in this study. Here we revealed a new mechanism by which AR-V7 cooperates with PIP5K1α and CDK1 to promote growth and invasion of PCa cells. AR-V7 physically interacts with PIP5K1α and CDK1 predominantly in the nucleus through formation of protein complexes. In PCa cells with PTEN mutations, AR-V7 is able to increase activity of pAKT. ISA-2011B blocks deregulated PIP5K1α/AKT activity and disrupt AR-V7/CDK1/PIP5K1α complexes in the nuclear and cytoplasmic compartment of PTEN-negative cells. The effect of ISA-2011B on AR may be mediated through CDK1, which is an upstream regulator and co-factor of both AR and AR-V7. The combination of ISA-2011B and enzalutamide synergistically inhibits deregulated AR and AR-V7 pathways in PCa cells.

In the present study, we show that AR-V7 physically interacts with PIP5K1α through formation of protein-protein complexes. We treated 22Rv1 cells with a proteasome inhibitor, MG132 to prevent proteins from degradation by proteasome-mediated pathways. PIP5K1α protein, but not AR-V7 is no longer degraded in the presence of MG132, suggesting that PIP5K1α protein stabilization is regulated by proteasome-dependent pathways. In the presence of MG132, ISA-2011B was unable to inhibit either PIP5K1α or AR-V7. Our finding suggests that the stability of AR-V7 protein is dependent on its complex-formation with PIP5K1α. Thus, the decrease in AR-V7 expression observed in ISA-2011B-treated cells is a result of disruption of stabilization of PIP5K1α/AR-V7 complexes (Figure 8). The precise molecular and cellular mechanisms involving the interplay among AR-V7, PIP5K1α and their functional targets will require further investigation.

In the present study, we show that ISA-2011B significantly suppresses growth of castration-resistant 22Rv1 tumors overexpressing AR-V7 in xenograft mice. Our data provide evidence suggesting that the effect of ISA-2011B on growth of 22Rv1 tumors is specific, and ISA-2011B acts on disruption of PIP5K1α/AR-V7 complexes, leading to degradation of AR-V7. In addition to its role as a potential co-factor to AR-V7 through complex-formation, PIP5K1α has a major role as a lipid kinase, and acts on PI3K/AKT/PTEN pathways [16]. As shown in our previous reported study, PIP5K1α promotes invasiveness of PCa cells that lacks functional PTEN and AR-V7^15^. In the absence of functional PTEN, PIP5K1α induces elevated activation of AKT and promotes PC3 tumor growth in xenograft mice [15]. In the present study, we further show that the effect of ISA-2011B on PI3K/AKT cascades is comparable to that of LY294002, an inhibitor of PI3K/AKT in PTEN-negative LNCaP cells. In contrast to LY294002 that is un-related to AR signaling, ISA-2011B has a strong inhibitory effect on AR-V7 and AR. We further show that the inhibitory effect of ISA-2011B on AR-V7 or AR is also in part dependent on CDK1, and this finding is in agreement with the previous reported study [15]. Thus, ISA-2011B has a potential therapeutic value for treatment of PCa harboring AR-V7-related resistance to anti-androgen therapy.

In the present study, we demonstrate that combination of ISA-2011B and enzalutmide have additive inhibitory effects on AR signalling in PCa. The second-generation anti-androgen enzalutamide represents an improvement in therapy options for late stage metastatic CRPC, however enzalutamide-treated PCa cells rapidly develops treatment resistance, most likely attributed to the constitutively active AR-V7 [11, 25]. Here, we show that treatment of PCa cells with enzalutamide diminishes cytoplasmic AR in 22Rv1 cells, suggesting that enzalutamide impairs agonist binding whereas ISA-2011B eliminates the nuclear AR expression, through PIP5K1α and CDK1. As AR interacts with PIP5K1α and CDK1, its activity in the nucleus is likely dependent in part on PIP5K1α and CDK1 [15]. The combination of ISA-2011B and enzalutamide synergistically inhibits deregulated AR and AR-V7 pathways in PCa cells. Our data suggest a compelling mechanism to circumvent enzalutamide treatment.

Taken together, our study identifies novel cooperative mechanisms involving PIP5K1α, AR-V7, CDK1 and AR, which drive tumor progression and contribute to enzalutamide resistance. Our study suggests that ISA-2011B and enzalutamide together can suppress tumor progression and metastasis through specifically blocking multiple pathways including deregulated AR pathways and elevated level of AR-V7 signaling complexes. Our findings provide new insights into the targeted therapeutic approaches to overcome resistance of PCa to enzalutamide treatment.

## MATERIALS AND METHODS

### Tissue Specimens, Tissue Microarrays and mRNA expression data

Tissue microarrays (TMAs) containing primary PCa (n=17) and metastatic PCa lesions in distant organs such as lymph node, lung, liver, and bone (n=43) from 14 PCa patients, and benign prostatic hyperplasia (BPH) (n=48) *vs*. matched PCa tissues (n=48) from 48 patients were constructed at Department of Clinical Pathology and Cytology, Skåne University Hospital, Malmö. The second TMA set that contained BPH and PCa tissues from 48 patients was purchased from Pantomics Inc. (Richmond, CA). The mRNA expression data of PIP5K1A and PTEN, and AR-V7 status was extracted from the dataset in the cBioPortal database, n= 333 tumors from 425 available cases in a prostate cancer patient cohort [26]. The detailed information of the data is available at https://tcga-data.nci.nih.gov/docs/publications/prad_2015/. IlluminaGA RNASeq and IlluminaHiseq RNASeq were analyzed [26]. The study was approved by the Ethics Committee at Lund University and Region Skåne in Sweden. The Helsinki Declaration of Human Rights was strictly observed.

### Immunohistochemical Analysis

Immunohistochemistry on tumor tissue microarrays was performed as previously described [27]. The staining procedure was performed using a semiautomatic staining machine (Ventana ES, Ventana Inc., Tucson, AZ). The sections were viewed under an Olympus BX51 microscope at magnification of 20X or 40X. Microphotographs were taken by using a high resolution scanner (ScanscopeCS, Aperio, Vista, CA). The specimens were evaluated and scored by four different scientists, including a pathologist. The staining intensity was scored as 0 (negative), 1 (weakly positive), 2 (moderate positive), 3 (strongly or very strongly positive) using an arbitrary semi-quantitative scale. The Gleason score 6 was used as cut off to define low *vs.* high grade.

### Cell Culturing and Treatments

The PNT1A cell line was purchased from Sigma Aldrich (Stockholm). LNCaP, 22Rv1and PC-3 were purchased from American Type Culture Collection (Manassas, VA) and were maintained in phenol red-containing RPMI-1640 medium supplemented with 10% fetal bovine serum (FBS), 1% penicillin-streptomycin-neomycin and 2 mM L-Glutamine (PAA Laboratories, GmbH, Austria). For treatment, cells were grown in phenol red-free RPMI-1640 medium containing 10% charcoal stripped-serum for 24 hours and then were treated with drugs alone or in combination for 24 hours or 48 hours. Enzalutamide at 5 μM or ISA-2011B at 20 μM or 50 μM final concentrations or solvent DMSO 1% was used. For treatment of 22Rv1 cells with MG132, a proteasome inhibitor, cells were treated with MG132 at 1 μM. For combination treatment of MG132 and ISA-2011B, cells were pre-treated with MG132 for 30 min at 1 μM prior to treatment of ISA-2011B.

### Plasmids, Stable Transfection, and siRNA Knockdowns

pLPS3-EGFP vector containing full-length human PIP5K1α cDNA or control empty vector were used as previously described [15]. pEGFP vector containing full-length AR-V7 and pEGFP vector were used as previously described [7]. pCMV-AR containing full-length AR and pCMV control vectors were kindly provided by Dr. Yvonne Giwercman at Department of Translational Medicine, Lund University, Sweden. Transient transfection was performed using Lipofectamine® 2000 transfection reagent (Life Technologies, Paisley, UK) according to the manufacturer's instructions. SiRNAs against *CDK1* or siRNA negative control duplex were purchased (VWR International Inc. Stockholm). SiRNAs (50nM) were transfected into 1 × 10^5^ PCa cells using Transfection Reagent TransIT-TKO® according to manufacturers’ protocol (Mirus Bio LCC). After introduction of respective siRNA complexes into PCa cells, cells were then collected after 24, 48 and 72 hours post-transfection.

### Mouse Models of Human Xenograft Tumors and Treatment

All animal studies were approved by the Swedish Regional Ethical Animal Welfare Committee. Athymic NMRI nude male mice (n=6 per experiment group) aged 8–12 weeks and weighing 25-27 grams each (Taconic Europe, Lille Skensved, Denmark) were used in each experimental setting. 2×10^6^ cells/mouse in 100 μl sterile vehicle solution were implanted subcutaneously into the flank. Tumor diameters were measured using calipers, and volumes were calculated using the equation (a x b^2^/2), where a and b represent the larger and smaller diameters, respectively. Tumors were grown to mean volume of 100-150 mm^3^. Xenograft mice with tumors were randomized (n=6 mice per group) and treatment of xenograft mice with vehicle control, ISA-2011B (40 mg/Kg) was initiated, and treatment for every second day was lasted for 15 days. The weights of mice were measured regularly and survival followed. Tumor samples were collected post-mortem and used for immunoblotting and immuno-histochemical analyses.

### Proliferation Assay

Proliferation of transfected PNT1A cells overexpressing AR-V7 or control vectors were determined using MTS proliferation assays (Promega Biotech) according to manufacturer's protocol. Cells at 5 × 10^3^/well were cultured in 96-well plates for 48 hours. MTS incorporation into the DNA was determined by measuring the absorbance on Infinite® M200 multimode microplate reader (Tecan Sunrise™).

### Immunoblot analysis and source of antibodies

The cells or tumor tissues were harvested and lysed in ice-cold RIPA buffer. Protein (20-30 μg) were separated with 12% SDS-PAGE gels and transferred onto nitrocellulose membranes. Signals were visualized using the Enhanced ChemiLuminescence detection system (Pierce, Rockford, USA) and documented with an AlphaImager CCD system. Densitometric quantification of immunoblots was performed by the ImageJ Image Analysis Software (NIH, Baltimore, USA) and represented as fold change relative to control and was normalized relative to actin or GAPDH bands. The following antibodies were used in this study: monoclonal antibodies against AR-V7 (produced in JL's lab) and AR-V7 (Abcam, Cambridge, UK), PIP5K1α (Proteintech Inc., and Cell Signaling technology), Phosphor-473 AKT (Cell Signaling technology and Santa Cruz Biotechnology), p27 (Cell signaling technology, Danvers, MA), androgen receptor AR (N20) and P27, Cyclin A2 and anti-GAPDH (Santa Cruz Biotechnology Inc. CA), PSA and Ki-67 (DAKO, Glostrup, Denmark), MMP-9 (Abcam, Cambridge, UK) and anti β-Actin (MP Biochemicals, Illkirch, France) cyclin E (Upstate Inc.) and Cdk1 (BD Transduction Lab Inc.) were used. Secondary antibodies: HRP-conjugated anti-mouse IgG and anti-rabbit IgG (GE Healthcare).

### Subcellular fractionation and Immunoprecipitation

Subcellular fractionation was prepared as previously described [28]. For obtaining the nuclear fraction, the pellets were incubated in the ice-cold nuclei isolation buffer (10 mM HEPES pH 7.9, 1,5 mM MgCl_2_, 10 mM KCl, 0.5 mM DTT, 1 % Triton X-100, 15 % protease inhibitor cocktail Complete Mini, 1 mM PMSF). After the separation steps, the nuclear and cytoplasmic fractions were obtained and subjected to immunoblot analysis. Antibodies against β-tubulin and Lamin B were used to validate the purity of the cytoplasmic and nuclear fractions, respectively. For immunoprecipitation (IP) assay, antibody against PIP5K1α was used to pull down the immunocomplexes, and antibody to IgG (BD Biosciences, San Jose, CA, USA) was used as a negative control. 500 μg of freshly prepared protein lysates were incubated with the antibody of interests and 30 μl of G-sepharose beads (GE Healthcare) for 3 hours at 4°C. The samples were then washed in RIPA buffer and subjected to immunoblot analysis.

### Immunofluorescence analysis

PCa cells were grown on the glass coverslips in phenol red-free RPMI-1640 medium containing 10% charcoal stripped-serum for 24 hours and were then treated with the indicated drugs for 24 hours. Cells were fixed with 4% paraformaldehyde in PBS. For blocking background staining from nonspecific interactions, Image-iT™ FX signal enhancer (Molecular Probes, Inc) was used. Primary antibodies against Phosphor-473 AKT, AR and AR-V7 were used. The secondary antibodies including rabbit anti-donkey conjugated to Rhodamine (Chemicon/Millipore International Inc, Temecula, CA, USA) or anti-goat conjugated to FITC antibodies at 1:200 and goat anti-rabbit Alexa Fluor 488 at 1:500 (Invitrogen, Stockholm, Sweden) were used. 4′,6-Diamidino-2-phenylindole counterstain (SERVA Electrophoresis GmbH, Heidelberg, Germany) was used to visualize cell nuclei. The slides were detected under an Olympus AX70 fluorescent microscope (Nikon DS-U1, Stockholm, Sweden). The software ACT2U was used (ACT2U version. 1.5, Stockholm, Sweden).

### Statistical Analysis

Tukey-test, ANOVA, Mann-Whitney test, student *t-test* and Spearman rank correlation tests were performed. All outcome variables are representative of at least three independent experiments. All statistical testes were two-sided, and *p* values less than 0.05 were considered to be statistical significant. Statistical software, Social Sciences software (SPSS, version 21, Chicago), was used.
